# Roles of Hepatic Innate and Innate-Like Lymphocytes in Nonalcoholic Steatohepatitis

**DOI:** 10.3389/fimmu.2020.01500

**Published:** 2020-07-16

**Authors:** Yongyan Chen, Zhigang Tian

**Affiliations:** ^1^Hefei National Laboratory for Physical Sciences at Microscale, The CAS Key Laboratory of Innate Immunity and Chronic Disease, Division of Molecular Medicine, School of Life Sciences, University of Science and Technology of China, Hefei, China; ^2^Institute of Immunology, University of Science and Technology of China, Hefei, China

**Keywords:** NASH, NK, ILC, NKT, γδT, MAIT

## Abstract

Nonalcoholic steatohepatitis (NASH), a progressive form of nonalcoholic fatty liver disease (NAFLD), is accompanied by steatosis, hepatocyte injury and liver inflammation, which has been a health problem in the world as one of the major high risk factors of cirrhosis and hepatocellular carcinoma (HCC). Complex immune responses involving T cells, B cells, Kupffer cells, monocytes, neutrophils, DCs and other innate lymphocytes account for the pathogenesis of NASH; however, the underlying mechanisms have not been clearly elucidated in detail. In the liver, innate and innate-like lymphocytes account for more than two-thirds of total lymphocytes and play an important role in maintaining the immune homeostasis. Therefore, their roles in the progression of NASH deserves investigation. In this review, we summarized murine NASH models for immunological studies, including the diet-induced NASH, chemical-induced NASH and genetic-induced NASH. The role of innate and innate-like lymphocytes including NK cells, ILCs, NKT, γδT and MAIT cells in the progression of NASH were elucidated. Further, the metabolic regulation of the innate immune response was addressed in consideration to explain the molecular mechanisms. Based on the findings of the reviewed studies, strategies of immune intervention are proposed to control the progression of NASH.

## Introduction

Nonalcoholic steatohepatitis (NASH) is a kind of liver disease, as the progressive form of nonalcoholic fatty liver disease (NAFLD), accompanied with steatosis, hepatocyte injury and liver inflammation as a consequence of disordered lipid metabolism in the liver. The prevalence of NASH is estimated to be between 1.5 and 6.5% globally, and up to 30% in individuals with fatty liver (hepatic steatosis). NASH was initially characterized by the hepatocyte death caused by lipotoxicity (1–3). NASH has been a global health challenge, since it is one of the major risk factors for the development of liver cirrhosis and hepatocellular carcinoma (HCC) (4, 5). As a multifactorial disease, the precise pathogenesis of NASH has not been fully elucidated, which is considered to be attributable to excessive fatty acids, oxidative stress, lipid peroxidation, mitochondrial dysfunction and some other extra-hepatic factors such as dysfunctional adipose tissue and altered gut microbiota (6). NAFLD/NASH is strongly associated with obesity, and the risk is generally increased with the degree of obesity (7, 8). Additionally, metabolic syndrome, especially the presence of type 2 diabetes mellitus (T2DM), insulin resistance, dyslipidemia and hypertension are risk factors of NAFLD progression (9). Signals derived from adipose tissue such as FFA, adipokine imbalance, pro-inflammatory cytokines TNF-α/IL-6 and insulin resistance promote the progression of NASH (10). The cross-talk between the gut and the liver including the microbiota modifications due to dysbiosis, intestinal permeability and metabolic endotoxemia are crucial events leading to NASH (10, 11). NAFLD is also featured with extra-hepatic manifestations as a risk factor for osteoporosis, thyroid dysfunction, chronic kidney disease, cardiovascular disease and colorectal cancer (12).

Accumulation of lipids and other lipid-derivatives in the liver affects hepatocytes, non-parenchymal hepatic cells and extra-hepatic cells. During the development of NASH, damaged hepatocytes evoke the immune responses by releasing various signals such as damage-associated molecular patterns (DAMPs) and pathogen-associated molecular patterns (PAMPs) to activate resident and recruited immune cells. Liver is an immunological organ that contains a large number of innate and adaptive immune cells; in particular, the largest populations of macrophages (Kupffer cells), natural killer (NK) cells, natural killer T (NKT) cells and γδT cells. Complex immune responses are involved in the pathogenesis of NASH including T cells, B cells, Kupffer cells (KCs), monocytes, neutrophils, dendritic cells (DCs) and other innate lymphocytes (13). Non-parenchymal hepatic cells with immune functions such as liver sinusoidal endothelial cells (LSECs) and hepatic stellate cells (HSCs), also participate in the development of NASH. With the further understanding of the composition of immune cells in the liver, including the discovery of novel cell subsets such as liver-resident NK cells, innate lymphoid cells (ILCs) and mucosa-associated invariant T (MAIT), the immunopathological mechanisms of NASH are necessary to be further analyzed and recognized. In this review, we discuss the current progress in the immunopathogenesis of NASH, mainly focusing on the hepatic innate and innate-like lymphocytes including NK cells, ILCs, NKT cells, γδT cells and MAIT cells. Based on the understanding of the immunopathogenesis of this disease, therapeutic strategies are proposed to control NASH.

## Murine NASH Models and Their Characteristics

Hepatic steatosis can be induced by fat accumulation, virus infection, medications or genetic disorders in humans. Steatohepatitis induced by factors other than alcohol consumption, virus infection and medication induction belongs to NAFLD. Accordingly, murine NASH models mimicking the pathological features of human NASH are defined as diet-induced NASH, chemical-induced NASH and genetic-induced NASH. Each kind of model exhibits distinct characteristics (Table 1).

**Table 1:** Characteristics for each murine model of NASH.

**NASH models**	**Inducer**	**Pathological characteristics**	**References**
**Diet-induced NASH**			
HFD	Tailor-made milk fat, anhydrous milk fat (21% w/w) and sucrose (34% w/w)	Obesity, insulin resistance Macrovesicular steatosis, hepatocellular ballooning Lobular inflammation, fibrosis (for 20 weeks)	(14)
HFHC	58% fat diet Drinking water with 42 g/L of carbohydrates (55% Fructose and 45% Sucrose by weight)	Significantly obese, insulin resistant Micro and macrovesicular steatosis Lobular inflammation, hepatocyte apoptosis Significant fibrosis (for 16 weeks)	(15)
HFHC*	15% fat and 1% cholesterol	Hypercholesterolemia and obesity Hepatic steatosis, substantial inflammation, perisinusoidal fibrosis, adipose tissue inflammation Reduced plasma adiponectin levels (for 30 weeks)	(16)
FFC	40% fat, 42 g/L fructose, and 0.2% cholesterol	Insulin resistance, steatosis, inflammation hepatocellular ballooning, progressive fibrosis (for 25 weeks)	(17)
WD	42% kcal from fat, 0.1% cholesterol with 23.1 g/L d-fructose +18.9 g/L d-glucose	Obesity, insulin resistance (for 8 weeks) Mild steatohepatitis and fibrosis (for 16–24 weeks) Tumors (for 52 weeks)	(18)
MCD	40% sucrose and 10% fat but deficient in methionine and choline	Weight loss, no insulin resistance Steatosis (mainly macrovesicular) (for 10 days) Inflammation and fibrosis (for 4 weeks) Increased fatty acid uptake, reduced VLDL secretion	(19, 20)
CD+HFD	Choline-deficient, 45% fat	Hepatic steatosis, NASH (6 months old) HCC (~25%) (12 months old)	(21)
HFMCD	Methionine and choline deficient diet supplemented with 60% fat	Not obese or insulin resistant Early-onset steatotic hepatitis, fibrosis (for 10 weeks)	(22)
**Chemical-induced NASH**			
CCL_4_/WD	21.1% fat, 41% sucrose, and 1.25% cholesterol and a high sugar solution (23.1g/L d-fructose and 18.9 g/L d-glucose) CCl_4_: 0.2 μL (0.32 μg)/g body weight	Attenuated insulin resistance Rapid progression of steatohepatitis Fibrosis (for 12 weeks) HCC (100%) (for 24 weeks)	(23)
STAM	STZ (200 μg, s.c.) 2 days after birth; 32% fat diet after 4 weeks of age	Decreased body weight, insulin resistance, hyperglycemia, steatosis (6 weeks old) Lobular inflammation (6–8 weeks old) Fibrosis (6–10 weeks old) A NASH-derived HCC model (16–20 weeks old)	(24, 25)
**Genetic-induced NASH**			
PTEN-/-	Hepatocyte-specific Pten-deficient Spontaneously	Macrovesicular steatosis, ballooning hepatocytes Lobular infiltration of inflammatory cells (10 weeks old) Perisinusoidal fibrosis (40 weeks old) Adenomas (100%), HCC (66%) (74–78 weeks old)	(26)
ob/ob or db/db	HFD or MCD; high-calorie diet or supplemented with iron	Insulin resistance Fatty liver, steatohepatitis (for 1–3 months)	(27, 28)
GNMT^−/−^	Spontaneously	Steatosis, steatohepatitis (3 months old) Cirrhosis and HCC (8 months old)	(29)
MC4R-KO	A high-fat diet	Obesity, insulin resistance, dyslipidemia (for 8 weeks) Steatohepatitis, inflammatory cell infiltration Hepatocyte ballooning, pericellular fibrosis Enhanced adipose tissue inflammation (for 20 weeks) Well-differentiated HCC (100%) (for 1 year)	(30)
ALR-L-KO	Liver-specific deletion of ALR Spontaneously	Steatosis, focal hepatocyte necrosis (2–4 weeks old) Prominent bile ductular proliferation Portal/periportal and lobular mixed inflammation (4–8 weeks old) HCC (nearly 60%) (1 year old)	(31)
Ldlr^−/−^	A high-fat-high-cholesterol diet (HFC) containing 21% milk butter, 0.2% cholesterol, 46% carbohydrates and 17% casein	Steatosis, hepatic inflammation Increased fibrosis and apoptosis (for 3 months)	(32)
APOE2KI	A western diet (17% casein, 0.3% DL-methionine, 34% sucrose, 14.5% cornstarch, 0.2% cholesterol, 5% cellulose, 7% CM 205B, 1% vit 200, 21% butter)	Inflammation (for 2 days) Steatosis, fibrosis Impaired VLDL secretion (for 4–7 days)	(33)
MUP-uPA	HFD (60% of calories are fat derived) starting at 6 weeks of age	Ballooning hepatocytes Extensive numerous immune infiltration Pericellular and bridging fibrosis (for 24 weeks) HCC (30%), adenomas (70%) (40 weeks old)	(34)

HFD, high fat diet; HFHC, high-fat high-carbohydrate; HFHC*, high fat, high cholesterol diet; FFC, high-fat, high-fructose and high-cholesterol diet; WD, western diet; MCD, methionine-choline deficient diet; CD-HFD, choline-deficient high-fat diet; HFMCD, high-fat methionine- and choline-deficient diet; STAM, a NASH-derived hepatocellular carcinoma (HCC) model; PTEN, phosphatase and tensin homolog; ob/ob, leptin deficient; db/db, leptin receptor deficient; GNMT, glycine N-methyltransferase; MC4R, melanocortin 4 receptor; ALR, augmenter of liver regeneration; LDL, Ldlr, low density lipoprotein receptor; APOE2KI, APOE2 knock-in; uPA, urokinase plasminogen activator.

### Diet-Induced NASH

Excessive free fatty acids (FFAs) that are not utilized by FFA oxidation and VLDL secretion, undergo re-esterification into TGs, and TG accumulation leads to hepatic steatosis. Therefore, high fat, high fructose or high cholesterol diets are used to generate NASH models in mice. Dietary factors also play an important role in shaping the composition and diversity of gut microbiota as demonstrated in animal models of NAFLD, which contributed to the progression of NASH (35).

High fat diets (HFD) are used to induce hepatic steatosis by increasing lipid delivery to the liver in animal models as a common method, but particularly without the occurrence of hepatocyte injury and liver inflammation in most strains of mice (34). However, HFD feeding can induce NASH in C57BL/6J mice with many pathophysiological features of human NASH (14). Interestingly, fructose consumption promotes the progression of NASH in mice, since high-fat high-carbohydrate (HFHC) diet could induce increased hepatic inflammation during the process of NASH (15). High cholesterol diet (HCD, 4% fat and 1% cholesterol) alone can only induced simple hepatic steatosis, with little inflammation. Dietary fat and cholesterol showed synergistic interaction in inducing NASH, as evidenced by the observation that high fat, high cholesterol diet (HFHC; 15% fat and 1% cholesterol) led to significant hepatic steatosis, liver inflammation and fibrosis (16). Mice fed by the FFC diet, a combination of high fat, fructose and cholesterol diet, recapitulated the features of human NASH better (17). A western diet (WD) mimicking fast food style diets, with high fat-fructose-cholesterol, was used to induce NASH with obesity and insulin resistance, but the NASH progression is not accompanied by severe steatohepatitis and fibrosis even the mice were fed for a long-term more than 24 weeks (18). A Western diet with liquid fructose (WDF) feeding mice showed significant steatohepatitis and fibrosis (22). HFDs induce the reductions in some types of gut microbiota including Bacteroidetes, Lactobacilli and Bifidobacteria, and the increase in Firmicutes, thus contributing to the progression of NAFLD partially through dysbiosis (36).

Additionally, choline as an essential nutrient for humans, participates in several metabolism pathways, which influence obesity and insulin resistance (37, 38). Overweight/obese subjects showed abnormal choline metabolism, and human subjects with NASH exhibited lower hepatic phosphatidylcholineto-phosphatidylethanolamine ratios compared to the healthy control (37). In the liver, choline is necessary to package and export TGs in VLDL and solubilize bile salts for secretion (37). Additionally, choline is required for intestinal bacterial metabolism, and in turn affects the composition of gut microbiome (38, 39). Humans fed with low-choline diets developed fatty liver and liver damage (40). Choline-deficient diet (CDD) and methionine-choline deficient (MCD) diet are used to generate NASH in mice (41). The choline-deficient L-amino acid-defined (CDAA) diet induces the same effects of the CDD (42). However, mice fed MCD diet or CD diet lose their body weight, without insulin resistance, although their liver tissues exhibit histological features of NASH (43). Thus, the combination of CD with HFD (CD+HFD) or MCD with HFD (HFMCD) has been used to induce NASH in mice with inhibition of weight loss (21, 22).

### Chemical-Induced NASH

Streptozotocin (STZ) is used to induce type 1 diabetes with the toxicity to pancreatic β cells. When mice administered with STZ were fed with HFD, they developed NASH with oxidative stress (24, 44). In the STAM model, low-dose STZ (200 μg) is administered to neonatal mice via a single subcutaneous injection immediately after birth, followed by feeding HFD (32% fat) at 4 weeks of age (25). Carbon tetrachloride (CCl_4_) can serve as an accelerator in inducing NASH through significantly promoting the activation of HSCs and up-regulating ductular reaction and hepatocyte proliferation (23). The combined use of CCL_4_ and WD led to a rapid progression of steatohepatitis, closely replicating the features of human NASH (23). A combination of chronic treatment with CCl_4_ and CDAA diet was also used to induce NASH with fibrosis and HCC (45).

### Genetic-Induced NASH

Leptin deficient mice (ob/ob) exhibit the accumulation of fat in the liver and severe insulin resistance, but rarely develop NASH. Additional stimulus such as HFD or MCD feeding has been shown to induce NASH development in ob/ob mice; however, the ob/ob mice model is of some limitation for the study of NASH because the levels of leptin are normal or elevated in NASH patients (27). Similarly, leptin receptor-deficient mice (db/db) also had fatty liver and insulin resistance, as well as the features of NASH when stimulated with high-calorie diet or supplemented with iron (28, 46).

Phosphatase and tensin homolog (*Pten*) is a tumor suppressor gene. Liver-specific *Pten* deficient mice develop NASH with hepatic steatosis, inflammation and progressive fibrosis, since *Pten* deficiency induced adipogenic and lipogenic gene expression in hepatocytes and altered glucose metabolism and insulin sensitivity, possibly associated with increased expression of transactivating factors peroxisome proliferator-activated receptor γ (PPARγ) and SREBP1c (26, 47). Glycine N-methyltransferase (GNMT) is the most important methyltransferase of the liver, which catabolizes S-adenosylmethionine (SAMe). GNMT-deficient mice developed spontaneous steatosis and progressed to NASH by spontaneously activating NK cells in the liver (29, 48). Melanocortin 4 receptor (MC4R) as a G protein-coupled receptor that expressed in the hypothalamic nuclei, regulates the food intake and body weight. MC4R-KO mice showed obesity, insulin resistance and dyslipidemia, and when fed HFD they developed steatohepatitis with infiltration of inflammatory cells, hepatocyte ballooning and liver peri-cellular fibrosis, similar to human NASH. Well-differentiated HCC was observed in the MC4R-KO mice with the incidence rate of 100% after a HFD feeding for 1 year (30). Augmenter of liver regeneration (ALR) as a hepatic growth factor, is critical for mitochondrial function, hepatocyte survival and lipid homeostasis. Mice with liver-specific deletion of ALR had excessive steatosis and hepatocyte apoptosis, and nearly 60% of these mice progressed to HCC (31, 49). Additionally, LDL receptor knock-out mice fed with a high-fat-high-cholesterol (HFC) diet showed steatosis, inflammation and increased apoptosis and hepatic fibrosis as a consequence of the increased sensitivity for oxidized LDL-induced inflammation, representing a highly suitable physiological model to study the onset of inflammation in NASH (32). APOE2 knock-in mice fed a western-type HFD developed NASH featured by steatosis and inflammation, due to impaired VLDL secretion and increased FFA synthesis and esterification or uptake (33). MUP-uPA mice with high amounts of urokinase plasminogen activator (uPA) specifically expressed in hepatocytes displayed classical features of human NASH and spontaneously progressed to HCC with additional treatment of HFD, in which hepatocyte ER stress was induced and consequently promoted the TNF production by inflammatory macrophages (34).

## Innate and Innate-Like Lymphocytes in NASH Development and Prognosis

**Figure 1:**
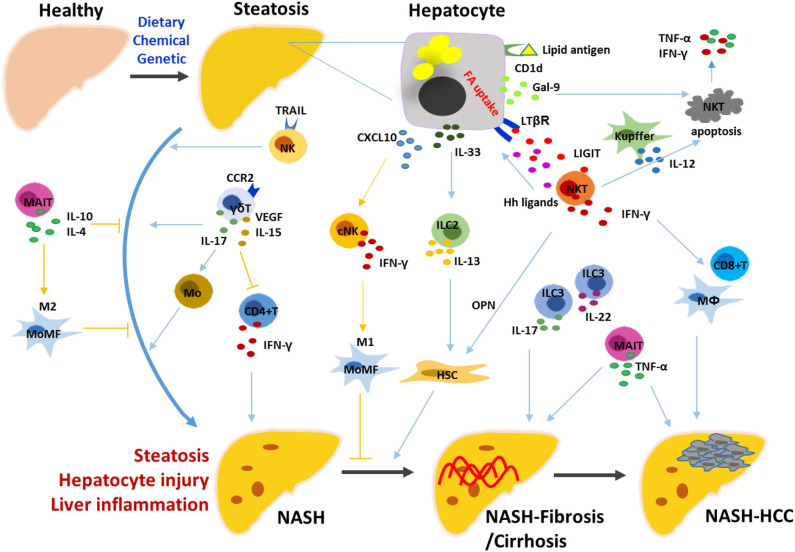
Mechanisms underlying the roles of innate and innate-like lymphocytes in the progression of NASH. Hepatic steatosis is induced by diet, chemical or genetic factors, characterized by the presence of hepatocytes with lipid accumulation. Stressed hepatocytes and non-parenchymal hepatic cells trigger the innate immune responses by releasing various signals. NK, ILC2/3, NKT, γδT, and MAIT cells are activated and exert their functions directly, or indirectly by linking the innate and adaptive immune cells including macrophages/monocytes, HSCs, CD4^+^ T and CD8^+^ T cells. NK, natural killer cell; NKT, natural killer T cell; ILC, innate lymphoid cell; MAIT, mucosal-associated invariant T cell; HSC, hepatic stellate cell; MoMF, monocyte-derived macrophage; Hh, hedgehog; OPN, osteopontin; Gal-9, galectin-9; LIGHT, LTβR ligand.

In the liver, innate and innate-like lymphocytes account for more than two-thirds of total lymphocytes and play a critical role in maintaining immune homeostasis. As a heterogeneous family, innate lymphoid cells (ILCs) are classified into groups 1, 2, and 3, including five cell populations, namely NK, ILC1 (group 1), ILC2 (group 2), ILC3 and lymphoid tissue-inducer (LTi) cells (group 3) (50–53). It has been described that NK cells are generated from NK cell precursors (NKPs); ILC1s, ILC2s, and ILC3s are generated from ILCPs (Lin^−^ CD7^+^ CD127^+^ CD117^+^ IL-1R1^+^ CD45RA^+^); LTi cells are generated from lymphoid tissue-inducer progenitors (LTiPs). Common innate lymphoid progenitors (CILPs) are responsible for the generation of NKPs and common helper innate lymphoid progenitor cells (CHILPs); and then CHILPs give rise to LTiPs and ILCPs (50, 54–56). ILC1s are tissue-resident cells with the expression of CD49a and CD69; therefore, liver-resident NK (LrNK) cells with CD49a positive were particularly difficult to be discriminated from them, making the one cell population with two titles (57, 58). Several types of innate-like T cells enriched in the liver include invariant NKT, MAIT, and γδ T cells. The roles of these innate and innate-like lymphocytes, including NK cells/ILC1s, other ILCs, NKT, γδT, and MAIT cells were elucidated in the development of NASH (Figure 1).

### Natural Killer Cells (NK) and ILC1s

In the liver, a large population of bulk NK cells represent ~30–50% of total lymphocytes in humans and 10% of total lymphocytes in mice (59). Two distinct subsets are termed as liver-resident NK (LrNK)/liver-resident ILC1s and conventional NK (cNK) cells with respect to their different origin, phenotype and function in the liver (60, 61). As described above, ILC1s develop from CHILPs that give rise to all members of ILCs but not NK cells; while NK cells develop form NKPs. Murine LrNK/ILC1s are characterized by a Lin^−^ CD45^+^ NK1.1^+^ NKp46^+^ CD49a^+^ CD49b^−^ phenotype independent on Eomes, but require the transcript factors T-bet, Hobbit, PLZF and AhR for their development; while NK cells are Lin^−^ CD45^+^ NK1.1^+^ NKp46^+^ CD49a^−^ CD49b^+^ and strictly require Eomes for their development (57). Recent studies have determined the respective contribution of these two NK subsets in NASH.

TRAIL-producing NK cells actively promoted the hepatic inflammation with strong cytotoxicity at the early stages of fatty liver disease in the absence of GNMT, suggesting they may contribute to the progression of NASH in the GNMT^−/−^ mice which spontaneously develop steatosis and progress to steatohepatitis, cirrhosis and HCC (48). However, increased number of CD49b^+^ NKp46^+^ cNK cells were demonstrated to prevent MCD-induced NASH progression to fibrosis by polarizing macrophage toward M1-like phenotypes, which depended on their production of IFN-γ but not granzyme-mediated cytotoxicity (62). Increased expression of CXCL10 early after MCD treatment accounted for the recruitment of cNK cells into the liver, resulting in the attenuated inflammatory infiltration of CD45^+^ CD11b^hi^ F4/80^int^ monocyte-derived macrophages (MoMFs) particularly Ly6C^lo^ subsets skewed toward M2 (63). The upregulation of expression levels of chemokines including CXCL10, CCL2 and CCL5 was demonstrated to be dependent on IL-15 signaling in response to HFD in hepatocytes, which accounted for the recruitment of immune cells (64). IL-15 deficiency mice showed attenuated NASH with significantly decreased number of NK cells in the liver (64). Additionally, given that leptin plays an important role in NK cell development and activation, the attenuated NK cells may contribute to the development of NASH in db/db mice with leptin receptor deficiency (28, 65).

LrNK/liver ILC1s are T-bet^+^ Eomes^−^ CD49b^−^ CD49a^+^ TRAIL^+^ with the potent ability to produce cytokines such as IFN-γ, TNF-α, IL-2, and GM-CSF in mice (56, 66). During the progression of MCD-induced NASH, the loss of CD49a^+^ NK1.1^+^ ILC1s was found to be induced in the liver, and the function of CD49a^+^ NK1.1^+^ ILC1s could not be precisely determined (62). Noticeably, ILC1s were enriched for the expression of inhibitory receptor CD200r1; while little to no expression of CD200r1 was observed on peripheral NK cells, and CD200r1 was stable to identify ILC1s during MCMV-induced inflammation, suggesting that CD200r1 will be a more useful marker to accurately track the ILC1s during inflammation (67). Adipose-resident ILC1s/trNK-derived IFN-γ triggered pro-inflammatory macrophage polarization and promoted insulin resistance in response to adipocyte stress induced by a high-fat diet (68, 69). At steady state, enriched adipose type 1 ILCs including circulating mature NK cells, tissue-resident iNK and CD49a^+^ mixed ILC1 subsets, killed adipose tissue macrophages (ATMs) to regulate ATM homeostasis, but the killing ability was impaired in obesity, contributing to the skewing toward pro-inflammatory macrophages and their accumulation in adipose (70). The conversion of effector NK cells (CD49a^−^CD49b^+^ Eomes^+^) was demonstrated into ILC1s including intermediate ILC1 (intILC1) (CD49a^+^ CD49b^+^ Eomes^+^) and ILC1 (CD49a^+^ CD49b^−^ Eomes^int^) populations that were not capable of restraining tumor growth and metastasis through TGF-β signaling in the tumor microenvironment (71). Whether these adipose ILC1-like cells undergo NK cell or ILC1 conversion need further investigation. In the obese liver, the conversion of NK cells toward ILC1-like cells with increased expressions of CD200r1 and CD49a was observed, partially mediated by the higher expression of TGF-β (72). The reduction in NK cell cytotoxicity due to the shift toward ILC1 characteristics showed protective roles in the progression of NASH (72). Additionally, lipids not only accumulate in hepatocytes but also in immune cells, suggesting metabolic reprogramming of NK cells may be involved in the pathogenesis of NASH. Obesity induced robust PPAR-driven lipid accumulation in NK cells, leading to impaired mTOR signaling and complete “paralysis” of their cellular metabolism and effector molecule expression, as demonstrated by the blunted anti-tumor responses (73). Involvement of liver-resident ILC1s in the development of NASH may be related to the inducing factors and pathological features of the disease.

In human NASH patients, hepatic CXCL9, CXCL10, CXCL11, CCL3, CCL4, and CCL5 levels were markedly higher than that of the healthy controls and the level of circulating CXCL10 was correlated with the severity of lobular inflammation (74, 75). These chemokines have the chemotactic activity of NK cells; however, the frequency of hepatic NK cells remained unaffected by clinical stage of NASH. The frequency and number of peripheral NK cells also remained unaltered in NASH patients. Only the phenotype of both CD56^bright^ and CD56^dim^ NK cells in the peripheral blood showed an alteration with much higher levels of NKG2D expressed on their surface (76). Notably, the number of hepatic NK cells was significantly increased in NASH patients with obesity compared with that in the healthy controls, along with higher expression levels of NKG2D and MICA/B in the liver, suggesting that NK cells may contribute to the progression of NASH through MICA/B (77). However, during obesity the number of peripheral NK cells increased and they were activated with elevated expressions of CD69 and Granzyme B, but had an impaired ability to respond and eliminate target tumor cells, which was highly related to the severity of obesity (78). The tissue-specific microenvironment is important to shape the local NK cell population, as shown by the significant differences in the composition of NK cells independently of disease status among liver, adipose tissue and peripheral blood. Studying the tissue-resident cell population is important to understand the pathogenesis of NASH (76).

In the human liver, unique resident NK cells were identified as CD49a^+^ CD56^bright^ NK cells, CXCR6^+^ NK cells or CD49e^−^ NK cells according to their some similarity to murine LrNK cells, respectively, (79–81). However, the presence of CD49a^+^ CD56^bright^ NK cells is unstable with a significant variation in the frequency in the human liver (79, 82). Recent studies showed that the ability of intrahepatic NK cells defined as CD45^+^ CD3^−^ CD56^+^ T-bet^hi^ Eomes^lo^ to degranulate and their killing activity was inversely correlated with the severity of NAFLD/NASH, and also the liver-resident NK cells defined as CD45^+^ CD3^−^ CD56^+^ T-bet^lo^ Eomes^hi^ exhibited decreased ability to degranulate (72). Given that the identification of human LrNK cells remains challenge, the roles of LrNK cells in the development of NASH are still unclear.

### Other Innate Lymphoid Cells: ILC2s and ILC3s

ILC2s are mostly localized in the barrier tissues such as gut, lung, skin and also liver, which express Gata-3 and RORα and secrete cytokines IL-5 and IL-13 (83). ILC3s are mostly concentrated in the gut, which express RORγt to drive the production of cytokines IL-17A and/or IL-22 (56, 84). The CD45^+^ Lin^−^ CD127^+^ CD16^−^ NKG2A^−^ CD161^+^ ILC population in the human liver can be subgrouped into four distinct populations: CD117^−^ NKp44^−^ CRTH2^−^ ILC1, CRTH2^+^ ILC2, and CRTH2^−^ CD117^+^ NKp44^−^ ILC3 and CRTH2^−^ CD117^+^ NKp44^+^ ILC3 cells, with a composition and phenotype significantly different dependent on age (85). In the adult liver, NKp44^−^ ILC3s accounted for the major population of total ILCs, while ILC2s represented a small population of ILCs (85). During the different steps of NAFLD/NASH development, poor studies evaluated the contribution of ILC2 and ILC3 subsets.

The frequency of ILC2s in the fibrotic liver was increased, which was correlated with the degree of fibrosis for patients; however, their phenotypes were similar to the ILC2s in nonfibrotic livers, with high expression level of CD69 and low expression level of CD62L, which were markedly distinct from the ILC2s in peripheral blood (85). The results indicated that the increased number of ILC2s in the fibrotic liver was not due to the recruitment of ILC2s from the peripheral blood, indicating the possible mechanisms in humans similar to the previous findings in mice. In a murine model of chemical-induced liver fibrosis, the expression of IL-33 was evaluated due to the chronic hepatocellular stress, and then IL-33 mediated the accumulation and activation of ILC2s through ST2-dependent signaling. Activated hepatic ILC2s produced IL-13, which in turn promoted hepatic fibrosis through triggering HSC activation (86). Additionally, IL-33 also could directly activate HSCs to drive hepatic fibrosis (87). In the human livers, IL-33 and thymic stromal lymphopoietin (TSLP) also promoted ILC2s to produce potentially profibrotic cytokine IL-13 (85). Adipose-resident ILC2s produced methionine-enkephalin peptides to regulate adipose function and metabolic homeostasis via promoting beige fat formation; and ILC2s-derived type 2 cytokines also played a critical role in the regulation of adipocyte precursor numbers and fate via the IL-4Rα in PDGFRα ^+^ adipocyte precursors (APs) to promote beige fat biogenesis (88, 89). However, ILC2 populations were dysregulated in white adipose tissue (WAT), as a conserved feature of obesity in mice and humans (88).

In human livers with fibrosis, the frequency of NKp44^−^ ILC3 was markedly decreased in accordance with the degree of disease. It was speculated that NKp44^−^ ILC3s in fibrotic livers plastically changed into ILC2s since the overall frequency of ILCs remained unaltered in fibrotic livers compared with that in the livers of healthy individuals (85). In a CCl_4_-induced mouse liver fibrosis model, larger numbers of IL-17A^+^ ILC3 and IL-22^+^ ILC3 subsets were presented in the liver and the pro-fibrotic effects of ILC3s were determined (90). However, localized IL-22 in the liver derived from T cells or some other undetermined cells was demonstrated as a survival factor for hepatocytes, preventing and repairing liver injury, accelerating liver regeneration, but promoting the susceptibility to tumor development (91–93). In obese mice, induction of IL-22 from ILCs was significantly impaired. Administration of IL-22 could reverse many of the metabolic symptoms via improving insulin sensitivity, preserving gut mucosal barrier, dampening chronic inflammation and regulating lipid metabolism in liver and adipose tissue (94).

### Natural Killer T Cells (NKT)

NKT cells are the major population of innate-like lymphocytes in the liver, which express both surface markers on NK cells (such as NK1.1 in mice and CD56 in humans) and semi-invariant (Vα14/Jα18/TCR β chains, including Vβ8.2, Vβ7 and Vβ2 in mice; Vα24/Jα18/Vβ11 in humans) or diverse TCRs, and are divided into two main subsets: invariant NKT (iNKT) and non-invariant NKT cells. iNKT cells are particularly enriched in the liver as liver-resident populations in mice and humans, which can recognize lipid antigens by CD1d (95). Their roles in the progression of NASH remain controversial.

iNKT cells were demonstrated to play a protective role against liver inflammation and liver injury progressing to fibrosis during the HFD-induced NASH, but not against lipid accumulation/steatosis (96, 97). In mice fed with a high fat or sucrose diet, increased apoptosis of NKT cells was induced in the liver, which resulted in the reduced NKT cells and promoted hepatic inflammation by excessive production of IFN-γ and TNF-α (98). The loss of NKT cells was dependent on KCs and IL-12 in hepatosteatosis (99). Additionally, the Gal-9/Tim-3 interaction induced Tim-3^+^ NKT cell apoptosis, which contributed to the depletion of NKT cells in the liver with HFD-induced steatosis (100). However, in Tim-3 knockout (KO) mice MCD-induced liver steatosis was significantly accelerated, which was demonstrated to be related to the activation of macrophages but not NKT cells (101). Notably, roles of NKT cells was oppositely determined by distinct populations of macrophages, since NKT cells activated by M2 macrophages inhibited mataflammation and insulin resistance by promoting Th2 responses in visceral adipose tissues (VATs) of obesity, while NKT cells activated by M1 macrophages exacerbated mataflammation and insulin resistance by promoting Th1 responses (102).

During MCD-induced NASH, NKT cells were accumulated via Hedgehog (Hh) pathway activation or IL-15 stimulation, and subsequently NKT cells promoted HSC activation to become myofibroblasts (MF) through production of osteopontin (OPN) and hedgehog (Hh) ligands (103–105). The hepatic recruitment and adhesion of NKT cells was related to the Hh-dependent production of CXCL16 and expression of VCAM-1 by immature ductular cells (103). In CD1d^−/−^ mice which lack NKT cells, the progressive liver fibrosis induced by MCD was prevented (103). In CDAA-induced and HFHC-induced murine NASH model, the presence of iNKT cells was also necessary for the steatosis, steatohepatitis and fibrosis (106, 107). Differential activation of iNKT cells skewed from predominantly NKT17 (IL-17^+^ iNKT and IL-22^+^ iNKT) to NKT1/NKT2 (IFNγ^+^ iNKT, IL-4^+^ iNKT and IL-13^+^ iNKT) played a pathogenic role in mediating the progression from steatosis to fibrosis by promoting the infiltration of CD8^+^ T cells and macrophages and the activation of HSCs (106). Further, by using a CD-HFD-induced NASH-driven HCC model, it was demonstrated that NKT cells primarily initiated steatosis via secreted LTβR ligand (LIGHT)-promoted hepatocyte FA uptake, and induced liver damage and HCC development cooperatively with CD8^+^ T cells (21).

In humans, the number of intrahepatic NKT cells was found to be significantly increased in the patients with moderate to severe steatosis, NASH or NSAH-cirrhosis (21, 103, 108). Notably, significantly enhanced expression level of LIGHT was found in the livers of NASH patients, and NKT cells were shown to be one of the major sources of LIGHT (21). In human NASH livers with advanced fibrosis, higher levels of OPN and Hh ligands were found compared with those with early fibrosis, and the levels were correlated with fibrosis severity, indicating the role of NKT cells-derived OPN in promoting fibrogenesis during NASH (105). The frequencies of peripheral CXCR3^+^ IFN-γ^+^ T-bet^+^ and IL-17A^+^ iNKT cells were also increased in NASH patients in comparison with those in non-alcoholic fatty liver (NAFL) patients or healthy controls (106).

### γδT Cells

γδT cells are a distinct subset of CD3^+^ T cells that express γδ TCRs. In the adult liver, γδT cells represent a frequency of 3–5% of the total hepatic lymphocytes, which is ~5–10-fold greater than that in other tissues and organs (109). Hepatic γδT cells are liver-resident cells that predominantly produce a high level of IL-17A, and their homeostasis is maintained by the gut microbiota in a lipid antigen/CD1d-dependent manner, including cell activation, survival and proliferation (110).

In HFD-fed or high-fat/high-carbohydrate diet (HFHCD)-fed mice, significantly elevated numbers of γδT17 cells were present in the liver, which contributed to the steatohepatitis, liver damage, and glucose dysmetabolism through the production of IL-17A (110). Furthermore, the increase in hepatic γδT17 cells occurred only in the presence of gut microbiota during the progression of NASH (110). During HFD-induced obesity, adipose tissue-resident γδT cells promoted inflammatory response by regulating macrophages, and increased systemic insulin resistance (111). In steatohepatitis, γδT cells expanded and exhibited a distinctly activated phenotypes with decreased expression of Vγ1, CD27 and CD69, and with increased expression of programmed cell death-1 (PD-1), CD1d and CD36 (112). The recruitment of γδT cells, mainly IL-17^high^ Ly6C^−^ CD44^+^ γδT cells was in a CCR2-dependent manner in the liver with steatohepatitis, which resulted in promoting the influx of inflammatory monocytes and mitigating IFN-γ production in CD4^+^ T cells (112). The γδT cell-derived cytokines VEGF and IL-15, but not IL-17A in a CD1d-dependent manner were demonstrated to play the key role in orchestrating the programming of the adaptive immune responses during the progression of NASH (112). During the progression of NASH, hepatic γδT cells played pathogenic roles by connecting the innate and adaptive immune responses (113). In the healthy human liver, infiltrating γδT cells (CD27^lo/−^ Vδ2^−^) comprise populations of both clonally expanded circulating and tissue-resident γδT cells, and liver-resident Vδ1^+^ γδT cells (CD45RA^−^, CD69^+^, CXCR3^+^, CXCR6^+^, CD103^−^, IFN-γ^+^, TNF-α^+^) are phenotypically and functionally distinct from circulating Vδ1^+^ γδT cells (CD45RA^+^, CD103^−^, CX3CR1^+^, Granzyme^+^, Perforin^+^) (114). A distinct population of Vδ3^+^ γδT cells is enriched and may also be activated to produce IL-17A in CD1d-dependent manner (115, 116). The pathogenic role of hepatic γδT cells in NASH patients deserves further investigation.

### Mucosal-Associated Invariant T (MAIT) Cells

MAIT cells, as an innate-like T cell population, are usually identified as CD3^+^ CD4^−^ CD161^+^ Vα7.2^+^ lymphocytes in humans and CD3^+^ CD4^−^ CD161^+^ Vα19^+^ lymphocytes in mice, with a limited repertoire of β chain (predominantly Vβ 6 and Vβ 20) (117, 118). MAIT cells recognize vitamin B metabolites by the presentation with major histocompatibility complex (MHC) class I-like molecule MR1. MAIT cells, which are clearly enriched in human livers, constitute up to 20–50% of all intrahepatic T cells, and are predominantly located to bile ducts in the portal tracts (117, 119). MAIT cells display an effector and memory phenotype (CD45RA^−^, CD45RO^+^, CD44^hi^, CD62L^lo^, CD122^int^, CD127^hi^, CD95^hi^) and rapidly respond to antigen stimulation in a TCR-dependent manner and to cytokine stimulation (such as IL-12, IL-18) in a TCR-independent manner, to produce cytokines TNF-α, IFN-γ, and IL-17 and cytotoxic molecules granzyme B and perforin (117, 119).

In patients with obesity, preferentially recruited MAIT cells were found in adipose tissue and displayed a strong production of IL-17, which resulted in a significantly reduced frequency in the peripheral blood (118). In patients with NASH-related cirrhosis, although the frequency of circulating MAIT cells was reduced, these cells exhibited an activated phenotype; furthermore, the frequency of hepatic MAIT cells was also decreased, but these cells accumulated in the mesenchymal space within the fibrotic septa (120). The profibrogenic functions of MAIT cells were demonstrated in CCl_4_-induced liver fibrosis by using MAIT cell-deficient mice and Vα19 TCR Tg-mice with a 10-fold increase in the frequency of MAIT cells (120). MAIT cells were found to enhance fibrogenic functions of hepatic myofibroblasts by promoting their proliferation in an MR1-dependent manner and enhancing their pro-inflammatory properties through the production of TNF-α (120). However, in a MCD-induced NASH model, MAIT cells were enriched in the livers and found to be protective against liver inflammation through the production of IL-4 and IL-10 and the induction of anti-inflammatory M2 macrophages (121). These controversial results indicated that the precise role of MAIT cells in the pathogenesis of NASH requires further clarification.

## Metabolic Regulation of Innate Immune Responses

**Figure 2:**
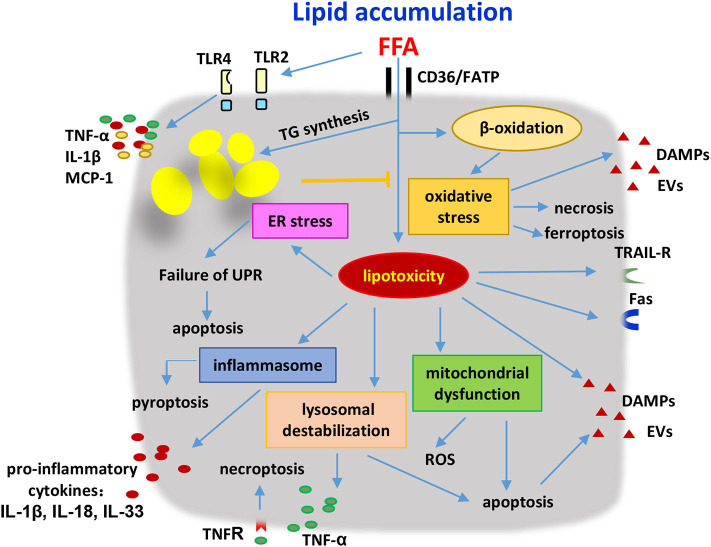
Hepatocytes were stressed or damaged by lipid accumulation and produced pathogenic mediators to induce the innate immune responses. Hepatocytes were induced to undergo apoptosis, necrosis, necroptosis and pyroptosis as a result of the lipotoxicity and oxidation stress. Lysosome destabilization, mitochondrial dysfunction, ER stress, inflammasome activation, and TLR signaling were involved in the development of metabolic disorders. Stressed or damaged hepatocytes secreted several kinds of pro-inflammatory cytokines including TNF-α, IL-1β, IL-18, IL-33, MCP-1, and produced DAMPs and EVs to activate the innate immune cells. Additionally, hepatocytes with lipid accumulation were sensitive to the cytotoxicity of immune cells due to the higher expression levels of death receptors on their surface. FFA, free fatty acids; FATP, fatty acid transport proteins; TG, triglycerides; ER, endoplasmic reticular; UPR, unfolded protein response; ROS, reactive oxygen species; DAMPs, damage-associated molecular patterns; EVs, extracellular vesicles; TRAIL-R, tumor necrosis factor-related apoptosis-inducing ligand receptor; TNF, tumor necrosis factor; IL, interleukin; MCP-1, monocyte chemoattractant protein 1.

In steatotic hepatocytes, lipids are major stored in the form of triglycerides (TG), and as several other lipid metabolites such as FFA, diacylglycerol (DAG), free cholesterol (FC), cholesterol ester (CE), ceramide, and phospholipid (122). Excessive accumulated lipids in hepatocytes may enter non-oxidative deleterious pathways, leading to hepatocyte damage. Here, metabolic regulation is addressed in consideration to explain the molecular mechanisms by which lipid accumulation in hepatocytes drives the activation of innate immune cells and the inflammatory response (Figure 2). Some studies also indicated that metabolic changes in innate and innate-like lymphocytes associated with NASH progression.

### Lipotoxicity

FFAs and other derived metabolites induce toxic effects known as lipotoxicity, which mediate the apoptosis, necrosis, necroptosis and pyroptosis of hepatocytes and then induce the inflammation in the liver (123–126). TG formation in hepatocytes plays a protective role by preventing FFA-induced damage to hepatocytes, since the inhibition of TG synthesis exacerbates liver damage and fibrosis although improves hepatic steatosis (127–129).

FFAs promoted hepatic lipotoxicity by stimulating TNF-α expression via the induction of lysosomal destabilization in a ctsb-dependent manner (130). FFAs sensitized hepatocytes with the high expression of TRAIL-receptor and the death receptor Fas (CD95) to the TRAIL-mediated and FasL-mediated apoptosis by immune cells (124). In humans, increased content of free cholesterol also sensitized hepatocytes to the apoptotic effects of TNF-α and Fas (131, 132). Saturated FAs as an endogenous danger molecule up-regulated the activation of inflammasome and also induced mitochondrial dysfunction to stimulate the production of reactive oxygen species (ROS) in hepatocytes, and furthermore FFAs and fragments as DAMPs derived from the damaged hepatocytes, activated and mobilized KCs, DCs and other innate lymphocytes by binding to TLR2, TLR4 and TLR9, resulting in the recruitment of neutrophils and lymphocytes and thus orchestrating the inflammatory response (124, 133). The elevated pro-inflammatory cytokines in the fatty livers including TNF-α, IL-1β, IL-18, IL-33, and MCP-1 activate and regulate NK cells, ILCs and other innate-like lymphocytes to participate in the pathogenesis. Additionally, in obesity lipid accumulation in NK cells via PPAR impaired their cellular metabolism and cytotoxicity (73).

FFAs also directly induced a hepatocyte endoplasmic reticular (ER) stress response, which led to failure of the unfolded protein response (UPR) and hepatocyte apoptosis (134–136). Inositol-requiring enzyme 1 alpha (IRE1α), protein kinase RNA (PKR)-like endoplasmic reticulum kinase (PERK) and activating transcription factor 6 (ATF6) are the ER stress sensors for lipids, and B-cell lymphoma 2 (BCL2)-associated X protein (Bax) inhibitor-1 (BI-1) is a negative regulator of the ER stress sensor. Reduced BI-1 led to the progression of NASH and type 2 diabetes, linked to the activation of NLRP3 inflammasome via up-regulating IRE1α endoribonuclease (RNase) signaling (137). ER stress-driven NF-κB activation promoted the secretion of inflammatory and chemotactic factors, involved in steatosis progression to NASH (138, 139). Additionally, ER stress decreased the expression of CD1d in hepatocytes, contributing to NKT cell dysregulation in fatty livers (140). However, ER stress in macrophages potently induced CD1d-dependent iNKT cell autoreactivity via IRE1α/PERK pathways, since macrophages undergoing ER stress generated the neutral lipids that were enriched and loaded onto CD1d to activate iNKT cells (141). HFD-enhanced ER stress in adipocytes suppressed Th2 cytokine expression, which modulated adipose tissue macrophage polarization toward M1 and mediated systemic insulin resistance (142). In T2D patients, ER stress was activated in NK cells, leading to the defects in the expression of the activating receptors NKG2D and NKp46 (143). Regulation of ER stress on the activity and function of innate lymphocytes deserves further investigation during the progression of NASH.

Further, hepatocytes exposed to lipotoxic stress released several types of extracellular vesicles (EVs) as pathogenic mediators to activate immune cells and promote inflammation, including exosomes, microvesicles and apoptotic bodies (126). Thus, lipotoxicity along with the activation of innate immune cells is the major driver of NASH.

### Oxidative Stress

In addition to the formation of TG, FFAs can be catabolized through mitochondrial or peroxisomal β-oxidation in hepatocytes, which consequently lead to the production of oxygen free radicals, hydrogen peroxide and other lipid peroxidation products (144, 145). In hepatocytes with lipid accumulation, oxidative stress induced the release of DAMPs and EVs, and the production of antigens, to initiate the innate immune response and trigger the adaptive immune responses, respectively (104). Promoted Th1 activation by oxidative stress-induced antigens induced NKT cell recruitment into the liver through selectively upregulated IL-15, which participated in the progression of NASH (104). Recently, it was found that alkaline ceramidase 3 (ACER3) mediated palmitic-acid-induced oxidative stress and played the pathological role in NASH. Targeting ACER3 could alleviate the severity of NASH by suppressing the hepatocellular oxidative stress, thus attenuating early inflammation and fibrosis but not steatosis, demonstrated by the similar area of steatosis and significantly reduced number of inflammatory foci, lower expression levels of IL-6, TNF-α and TGF-β, and minor fibrosis in Acer3^−/−^ mice (146). Oxidized phospholipids (OxPLs) accumulated and induced oxidative stress and mitochondrial damage in NASH. Neutralization of OxPLs was effective to ameliorate NASH by decreasing the expression of a variety of inflammatory chemokines and cytokines such as *Ccr2, Ccr5, Ccl6, Cx3cr1, Cxcl14, Il1a*, reducing recruitment of monocyte-derived macrophages into the liver and promoting shift of Tim4^−^ to Tim4^+^ macrophages that mediated engulfment of apoptotic cells (147). Ferroptosis induced by lipid peroxidation was considered to play a major role in the development of NASH with increased expressions of IL-1β, IL-6, TNF-α, TGF-β, and MCP-1 (148, 149).

## Intervention Strategies Against NASH Based on Innate and Innate-Like Lymphocytes

The effective prevention and control of NASH remains a clinical challenge. As described above, stressed/damaged hepatocytes with lipid accumulation trigger innate immune responses through several pathways; furthermore, hepatic NK, ILCs, NKT, γδT, and MAIT cells are involved in the pathogenesis of NASH. Based on these findings, the development of immune-based interventions to control the progression of NASH has been proposed.

Anti-inflammatory agents targeting TNF-α signaling, such as a pan-caspase inhibitor emricasan and a methylxanthine derivative pentoxifyllline (PTX), were evaluated in clinical trials for NASH patients (150). However, emricasan treatment lowered serum ALT in the short-term but worsened liver fibrosis and hepatocyte ballooning due to alternative cell death in patients with NASH; and also no beneficial effect of emricasan was observed in NASH-related cirrhosis patients (151, 152). As described above, the contribution of NK cells and ILC1s was evaluated in the hepatocyte injury and liver inflammation during the progression of NASH, suggesting a strategies against NASH through targeting NK and ILC1s, especially the metabolic regulation (72, 153). Strategies targeting the IL-33/ILC2/beiging pathway may represent a new therapeutic approach to treat obesity and obesity-associated diseases (88). The therapeutic modulation of IL-33-dependent networks has also been demonstrated in hepatic inflammation and fibrosis (86). Given the critical role of CD1d in the activation and regulation of γδ T cells, approaches involving the targeting of CD1d and γδT cells may have therapeutic potential for the treatment of NASH (110). Treatment with the RAR-γ agonist Tazarotene was found to significantly reduce steatosis and fibrosis by inhibiting the proliferation and cytokine secretion of iNKT cells (106). Dietary glycine has been shown to prevent the progression of NASH by ameliorating oxidative stress and inflammatory infiltration involving NKT cells as well as M2 transformation of KCs in the liver (154). Efficiently reducing the numbers of intrahepatic CD8^+^ T and/or NKT cells, as well as preventing their cross-talk with hepatocytes by targeting LIGIT-LTβ R or the hedgehog pathway offers the potential to inhibit liver damage, NASH and HCC (21, 105).

## Conclusion

Liver is an immunologic organ that contains a large number of innate immune cells. Using murine NASH models, innate and innate-like lymphocytes, including NK cells, ILCs, NKT, γδT, and MAIT cells have been demonstrated to play critical roles in the progression of NASH. Other innate immune cells such as KCs, neutrophils and DCs have been described in detail elsewhere. Disordered lipid metabolism in hepatocytes initiates the innate immune responses. Additionally, extra-hepatic factors such as dysfunctional adipose tissue and altered gut microbiota, as well as extra-hepatic cells have also been found to be involved in the development of NASH. Further studies in NASH patients are necessary to confirm these findings. Continuous efforts toward understanding of the complex immune mechanisms underlying NASH development should offer new avenues for the management and prevention of this disease.

## Author Contributions

YC prepared the manuscript, table, and figures. ZT designed and gave guidance on the outline, and revised the manuscript. All authors contributed to the article and approved the submitted version.

## Conflict of Interest

The authors declare that the research was conducted in the absence of any commercial or financial relationships that could be construed as a potential conflict of interest.
